# K-core robustness in ecological and financial networks

**DOI:** 10.1038/s41598-020-59959-4

**Published:** 2020-02-25

**Authors:** Kate Burleson-Lesser, Flaviano Morone, Maria S. Tomassone, Hernán A. Makse

**Affiliations:** 1 Levich Institute and Physics Department, City College of New York, New York, 10031 New York USA; 2 The Graduate Center at the City University of New York, New York, 10016 New York USA; 3 Rutgers Department of Chemical and Biochemical Engineering, Rutgers University, Piscataway, 08854 New Jersey USA

**Keywords:** Mathematics and computing, Physics

## Abstract

In many real-world networks, the ability to withstand targeted or global attacks; extinctions; or shocks is vital to the survival of the network itself, and of dependent structures such as economies (for financial networks) or even the planet (for ecosystems). Previous attempts to characterise robustness include nestedness of mutualistic networks or exploration of degree distribution. In this work we present a new approach for characterising the stability and robustness of networks with all-positive interactions by studying the distribution of the k-shell of the underlying network. We find that high occupancy of nodes in the inner and outer k-shells and low occupancy in the middle shells of financial and ecological networks (yielding a “U-shape” in a histogram of k-shell occupancy) provide resilience against both local targeted and global attacks. Investigation of this highly-populated core gives insights into the nature of a network (such as sharp transitions in the core composition of the stock market from a mix of industries to domination by one or two in the mid-1990s) and allow predictions of future network stability, e.g., by monitoring populations of “core” species in an ecosystem or noting when stocks in the core-dominant sector begin to move in lock-step, presaging a dramatic move in the market. Moreover, this “U-shape” recalls core-periphery structure, seen in a wide range of networks including opinion and internet networks, suggesting that the “U-shaped” occupancy histogram and its implications for network health may indeed be universal.

## Introduction

Whether one examines ecological networks^1–3^, financial networks^4–7^, social networks^8,9^, neural networks^10,11^, or beyond, the identification of the features of these networks that characterise their robustness and resilience against external shocks is an important problem in network science today. The relation between network structure and network stability is essential to understanding why some networks survive in the face of global and random local changes, and why others do not. There have been many attempts to characterise what defines robustness in a network based on the features of the network, and the effects of this robustness on network dynamics^9,12–17^. Specifically, this has many applications in ecology^2,3,18–22^, where the robustness of an ecological network of species may determine the ability of that network to withstand environmental changes, and in finance^6,7^, where it is important to have a robust financial network in order that the economy does not collapse. Furthermore, by identifying the species or companies that are most integral to network robustness, we may discover markers of future network health. In the ecological case, these species are typically monitored to make sure that their numbers do not fall too low and thus endanger the health of the entire ecosystem; in the case of the stock market, the financial sectors composed of these most-important stocks are usually monitored for lock-step correlations in returns, which is a warning sign of economic turbulence^23^. This “lock-step” behaviour has become especially prevalent—and dangerous—since the 1990s, when financial deregulation led banks to repeatedly merge until the majority of money invested in the stock market was controlled by a few very powerful institutions^24–28^. Moreover, the use of automated trading algorithms has introduced further homogenisation, as these few banks now tend to buy or sell stocks based upon similar “advice”^29–31^.

Due to the importance of characterising the robustness of complex systems, there have been many attempts to introduce measures of robustness based on structural properties of the network. In supply networks, for example, it has been shown that a network with a scale-free degree distribution will be more resilient than a network with a centralised or block-diagonal structure^12^. Similarly, studies of the robustness of the Internet have shown that, for large networks whose degree distributions are power-laws, an exponent less than or equal to 3 will lead to increased resilience^13^. Despite the amount of work done on the resilience of networks, most of the published research on this topic has concentrated on experimental application of complex network theory to existing networks, or examination of the structures (such as nestedness^19,21^ or core-periphery^14–16^) of existing networks. None of the previous work has focused on a theoretical formalism aimed at finding the relationship of the occupancy of the k-core to the resilience of a network under both global and random attack. In this paper, we propose a new approach to quantify the resilience of the network and demonstrate that the occupancy of the k-core^32–35^ can be linked to the resilience of dynamical mutualistic ecological networks and networks of stocks in the S&P 500 under both random and global attack. We focus on a particular ecosystem, plant-pollinator interactions, but our results can potentially be applied to other mutualistic ecosystems. Following a k-core decomposition of the ecological and financial networks used in^18^, we find a quite universal U-shaped occupancy curve in their k-shells: the outer shells and inner core are highly occupied while the intermediate shells are not. This is reminiscent of the core-periphery structure found in the internet, opinion networks, and others^14–16^. The mutually-beneficial nature of interactions among members of the ecosystem means that, when quantified, these interactions are positive. For example, predator-prey interactions, would be positive for the predator but decidedly negative for the prey, while competitive interactions are negative for all involved species; in other biological systems such as neural and genetic networks, these negative interactions represent inhibitory or repressing interactions between neurons or genes. The financial systems discussed in this paper have all-positive interactions as well. This universal k-core structure provides robustness to the system against global changes such as climate change while providing resilience against random local attacks on the network’s species.

Previous work in the field of complex networks relates ecosystems and financial systems, and the latter are occasionally referred to as “financial ecosystems”^6,36^. Here, we consider both real and “financial” ecosystems as networks of interacting species/stocks, with each species/stock as a node in this network, and an interaction between a pair of species/stocks taken as an edge, or link between the two nodes. The ecosystems are mutualistic— members interact in such a way that each derives some benefit from being a part of the network— and those members that contribute as well as derive a benefit are the most important to the integrity of the system and are called “symbionts”^7,19,37–39^. We examine the network structure via k-shell decomposition^32–35^ and simulate “attacks” which would remove nodes (species) from the network in order to probe robustness. Compared to a set of random networks, we find that the structure of these networks differs in a way that renders them considerably more robust against random attack: they have more nodes in the outer k-shells of the network, i.e., more nodes that have few connexions or are connected mainly (or only) to nodes with few connexions. In the parlance of ecology, these species are commensalists, or species that depend upon other species in the ecosystem more than they themselves are depended upon. The chances are thus higher that one of these outer nodes (commensalists) will be removed rather than one of the strongly interconnected central nodes (symbionts).

Furthermore, the mapping to the k-core dynamical model indicates that the inner shells, or maximum k-core, are most robust under global attack on the network. We are inspired by the result of^18^ which is further expanded upon in^11^ and suggests that network robustness increases with maximum-core occupancy. We prove that for maximal resilience under both random local and global attack, a network requires both the maximum k-core and outermost shells to have high occupancy, corresponding to the U-shape found in ecosystems and financial networks. We see that robustness in the face of random attacks and global changes increases as there are more species in both the inner core and outer shells. This implies that greater diversity leads to greater stability in ecological networks, rather than the opposite as it has been proposed in the works of R. May and collaborators^3,6,7^.

## Results

### Theoretical formalism

We will begin with a brief discussion of k-shell decomposition of a network. The k-shell decomposition is used to partition the network into hierarchically ordered sub-structures, by decomposing the network iteratively removing all the nodes with order smaller than the index of the current shell until no removing is possible^32^. We divide our networks into a series of concentric “shells,” from the outermost to the innermost or “core”^32,33,40^. Each node is assigned an index *k*, and nodes that have the same value of *k* constitute the k-shell. Following a series of iterative prunings based upon degree *k*, each node is assigned a shell based upon its ultimate degree. From here, one can learn valuable information about the network’s structure, such as the occupancy of each shell; the level of *k* at which the core exists; and the tipping point of collapse for the network *K*_*γ*_ (discussed in the following section)^18^.

We explain the algorithm in greater detail below:

1. Find all nodes with degree *k* = 1.
2. Remove these nodes and their links from the network.
3. Since there may now be new nodes that have degree *k* = 1, repeat steps **1** and **2** until there are no more nodes with degree *k* = 1 in the network; these nodes belong to the *k* = 1 shell, or *k*_*s*_ = 1.
4. Repeat the previous three steps for increasing values of *k* until all nodes in the network have been categorised as belonging to a k-shell.
5. Those nodes belonging to the innermost k-shell—*k*_*max*_—are considered as belonging to the "k-core” of the network.

**Figure 1:**
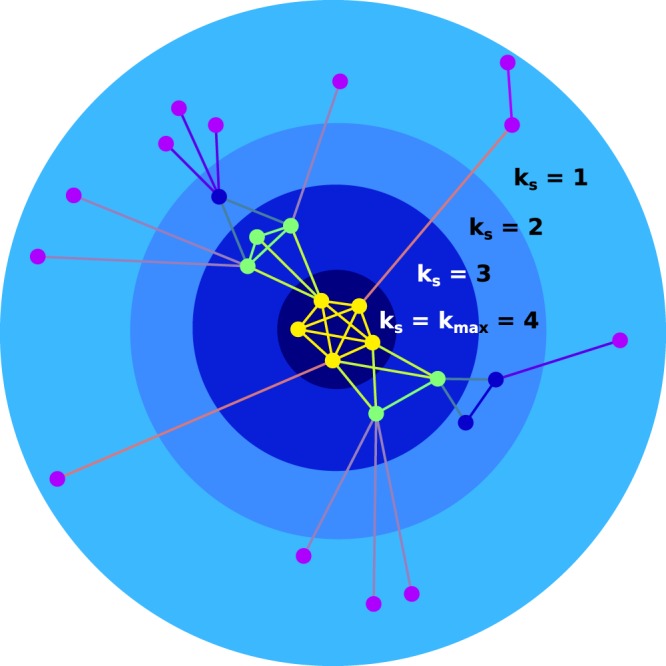
K-shell decomposition of an example network. The network shown here has undergone the k-shell decomposition described above. At each step of the algorithm, nodes with degree equal to or less than *k* are removed iteratively until none remain. These nodes are then said to belong to the *k*-th shell. The innermost shell *k*_*max*_, or the core of the network, contains the nodes with the highest degree even after the iterative pruning, which are the ones responsible for providing a “structure” to the network with their high interaction strengths. Those in the outermost shell (or shells, in larger networks) are vital for network survival under random attack, where a greater number of “expendable” nodes in the outer shells decreases the chances of a vital node being removed. The “U”-shaped k-shell occupancy levels described in this paper therefore lead to networks that are more robust under both global and local attacks.

The members of the network may then be grouped by the shells to which they belong. Figure 1 shows an example network divided into its constituent k-shells, with the innermost shell being the maximum core.

To justify the importance of a highly-occupied k-core for network resilience, we propose a simple model in Equation 1, the solution of which is based on the solution found in^18^ and shows that the high occupancy of the core protects against network collapse:

1 $${\dot{x}}_{i}(t)=-\,d{x}_{i}+\gamma {\sum }_{j=1}^{N}{A}_{ij}\frac{{x}_{j}^{n}}{{\alpha }^{n}+{x}_{j}^{n}}.$$

Given some network with adjacency matrix *A*_*ij*_ equal to 1 if species interact and 0 otherwise, ${\dot{x}}_{i}(t)$ as the density (population per unit area) of species *i*; *d* > 0 is the rate of species die-off; *α* is a half-saturation constant; *n*, the exponent of the Hill coefficient, governs the steepness of the functional response given by *H*_*n*_(*x*_*j*_, *α*) and is taken as being equal to or greater than 2^41,42^; and *γ* > 0 is the maximal interaction strength between pairs.

There is a nontrivial fixed point where each species in the system has some nonnegative density^18^. Logically, this is a possible solution because in actuality, a species cannot have a negative density. The average of all interactions at this fixed point will be a critical value *γ*_*c*_. In^18^, it is found that for average interactions *γ* above this value, the system survives at a nontrivial average fixed point, though if *γ* drops below *γ*_*c*_, the ecosystem becomes extinct, i.e., average density of species goes to 0.

Equations 1 apply to the ecological networks due to the mutualistic, or all-positive, nature of the interactions (as opposed to predator-prey or competitive networks, which contain negative interactions and require a more general theory than the k-core percolation shown in^18^). Furthermore, the financial networks, likened to an ecosystem by May and collaborators^6,36^, also have completely positive interactions *J*_*ij*_. (Financial networks, like ecosystems, evolve, albeit over a much shorter time scale. There is also an element of mutualism at play: a bank might lend to a steel plant, which ships raw materials to an automobile factory, and so forth).

Equations 1, then, are a model which predicts that the k-core is what determines the stability of the system. We do not claim that Eq. 1 can be applied directly to financial networks, but the k-core analysis is still valid, because it represents a network tool that can be applied to any network, independently of the validity of Eq. 1. Our study on the financial networks is based on the application of the occupancy of the k-shell in the same manner as for biological ecosystems.

The tipping point of the system can be determined by solving Equations 1 using the logic approximation of the Hill function (see^18^ for details) (*H*_*n*_(*x*_*j*_) ≈ Θ(*x*_*j*_ − *α*), exact as *n* → *∞*). This point is related to the maximum k-core of the network as shown in Eq. 4 for equations with all-positive interactions because here, any decrease in *γ* would cause an increase in ${K}_{{\gamma }_{c}}$. The logic approximation equals to 1 for *x* > 0, and 0 otherwise, meaning that the fixed point of Eq. 1 is given by

2 $${x}_{i}^{\ast }=\frac{\gamma }{d}{\sum }_{j=1}^{N}{A}_{ij}\Theta ({x}_{j}^{\ast }-\alpha ),i=,\ldots ,N$$

with uniform dynamical parameters chosen for simplicity. Doing a change of variables ${y}_{i}^{\ast }=\frac{{x}_{i}^{\ast }d}{\gamma }$, this becomes

3 $${y}_{i}^{\ast }={\sum }_{j=1}^{N}{A}_{ij}\Theta ({y}_{j}^{\ast }-{K}_{\gamma })$$

where the degree threshold for calculating the k-core is ${K}_{\gamma }=\frac{\alpha d}{\gamma }$. The tipping point ${K}_{{\gamma }_{c}}$ of the network—the point beyond which it collapses—is then

4 $${k}_{core}^{max}={K}_{{\gamma }_{c}}=\frac{\alpha d}{{\gamma }_{c}}.$$

Following this, the densities of all “species” would go to 0 (i.e., total network collapse) because the threshold for interactions would be too high to allow any mutualistic interactions between species. As the reduced densities ${y}_{i}^{\ast }$ can only take integer values from 1 to *k*_*i*_ (where *k*_*i*_ is the degree of species *i*), all species with *k*_*j*_ < *K*_*γ*_ are removed via the Heaviside function in Equations 3 so as to solve for ${y}_{i}^{\ast }$ at a threshold *K*_*γ*_^18^. Thus, the removal of any one species from the maximum k-core will cause all other species in the core now to have too few links to belong to that maximum core and it will collapse.

The remaining species now have a new degree $k{\prime}$; the network is checked again and species with $k{\prime}  < {K}_{\gamma }$ are removed, continuing until no species has degree less than *K*_*γ*_. This is the same algorithm used in k-shell decompostion^18,32,35^. The species left after this process, then, constitute the *K*_*γ*_-core. We showed in our previous publication^18^ that the reduced density of species *i*, ${y}_{i}^{\ast }$, is equal to the number of links *N*_*i*_(*K*_*γ*_) from species *i* to the core species: ${y}_{i}^{\ast }={N}_{i}({K}_{\gamma })$ (the nontrivial fixed point solution for species in the *K*_*γ*_-core)^18^. In this way, the network dynamics and the network structure are linked and the removal of links to the *K*_*γ*_-core leads to a total network collapse. The densities ${x}_{i}^{\ast }$ and reduced densities ${y}_{i}^{\ast }$ are greater than 0, so the number of links to the *K*_*γ*_-core must also be greater than 0. For average interactions *γ* with some value that forces *K*_*γ*_ to be larger than the maximum k-core of the network, there are no links to the maximum k-core; the network collapses to the extinct state ${x}_{i}^{\ast }=0$ with critical threshold ${K}_{{\gamma }_{c}}$. As a corollary of Eq. 1, the highly-occupied innermost k-shells are more resilient.

This result may also be applied to, among others^43,44^, financial systems which evolve dynamically^6^. While the time scale is much smaller than that which shapes an ecosystem, it cannot be denied that certain businesses and industries, and the economies which are centered around them, have continued to thrive while others have failed. The S&P 500 stock index is an example of such a system; the weaker performers are, over time, removed (as a species might go extinct from an ecosystem) while those businesses at the core of the network under a k-shell decomposition, which provide stability and robustness, have persisted over decades. If the weaker performers in the S&P 500 are also those at the periphery of the associated network, there is little disturbance in the economy as a whole when they are removed from the network of the S&P 500. Financial crises, analogous to an ecosystem being pushed to (or even past) the brink of collapse, occur when the businesses in or near the k-core begin to perform weakly and fail, thus endangering their presence in the stock index and potentially leading to the disappearance of nodes essential for the maintenance of network structure.

### Network structures: ecological and financial

We begin the analysis by constructing our networks, both ecological and financial.

#### Ecological network

The ecological data are found from field studies in which interactions between various species in mutualistic ecosystems (i.e., plants and pollinators) are recorded and enumerated^45^. For example, one field study might count the number of bees and wasps visiting various plants within a certain area over a certain period of time^46^. These networks are straightforward to construct: adjacency matrices where a value of 1 denotes some directed interaction between one species and another, and a value of 0 denotes no interaction. An interaction here is a visit by a pollinator to a given plant; the networks constructed are bipartite and directed^18^. As these are mutualistic systems, where the plant provides a benefit to the pollinator in the form of food and the pollinator helps the plant to disperse pollen, all of the interactions are nonnegative.

**Figure 2:**
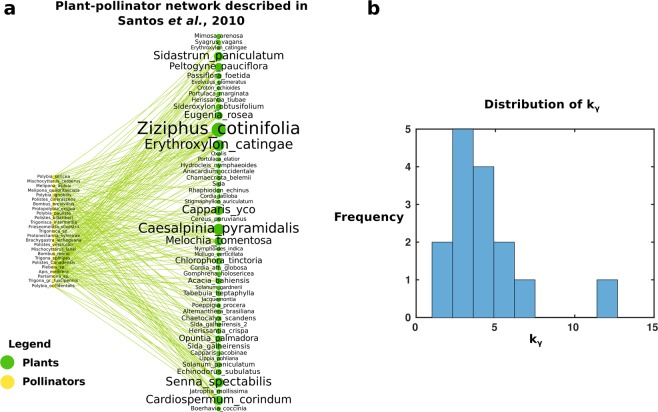
Example ecological network. (**a**) Schematic of a network built from the adjacency matrix of plant-pollinator interactions between a total of 76 plants and pollinators described in Santos *et al*.^46^ (**b**) the distribution of tipping points *K*_*γ*_ across all ecological networks considered in this work. Interactions in (**a**) are directed from the acting species (the pollinators) to the passive species (the plants); node size increases with in-degree. Green nodes represent plant species while yellow nodes represent pollinators.

Figure 2a shows as an example the ecological network studied by Santos, Aguiar, and Mello (2010)^46^. This plant-pollinator network is composed of the interactions of various species of social bees and social wasps in Bahia, Brazil with various types of plants. We display the network in a directed bipartite format, with the bees and wasps acting upon the plants by visiting them, following the convention of an interaction originating with an active party and performed upon a passive party^21^. Node size scales with in-degree, or the number of visits received by a given species of plant. Figure 2b shows the distribution of the parameter *K*_*γ*_ (the “tipping point” according to Eq. 4) for the 15 mutualistic plant-pollinator networks^46–59^ gotten from InteractionWeb^45^.

#### Financial network

In our examination of the set of financial data, we construct both pairwise correlation and pairwise interaction networks— *C*_*ij*_ and *J*_*ij*_, respectively^60,61^— using logarithmic returns of stocks belonging to the S&P 500. Given these time series of stock log-returns, we first calculate the pairwise Pearson correlations *C*_*ij*_ and then infer the pairwise interactions *J*_*ij*_ using maximisation of the log-likelihood via the Graphical Lasso algorithm^60,61^. A detailed explanation of each method can be found in the Supplementary Information, in “Correlation matrices” and “Interaction matrices.” The data covers a period of roughly 45 years (2 January 1970 to 5 November 2015)^62–65^ and is divided into overlapping windows of 100 days (see Methods section for further details).

**Figure 3:**
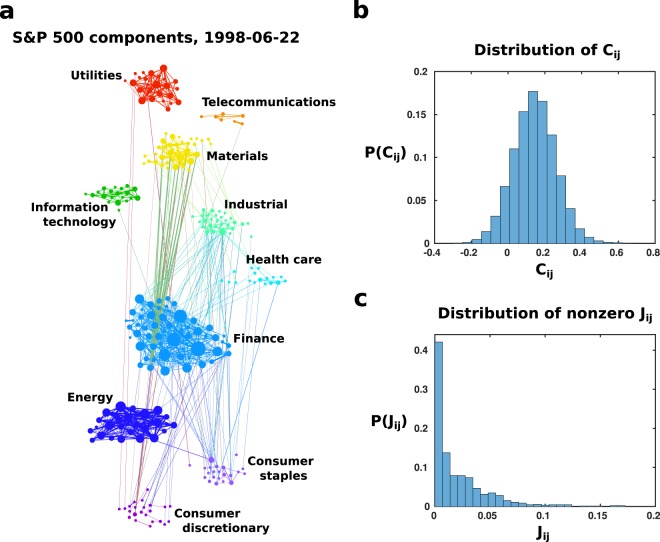
Example network of the S&P 500. (**a**) Schematic of the network built from pairwise interactions *J*_*ij*_ between 489 stocks in the 100-day window ending 22 June 1998. Nodes are colored according to the financial sector to which they belong, and node size increases with degree. Interactions are symmetric, i.e., *J*_*ij*_ = *J*_*ji*_. (**b**,**c**) show the distribution of pairwise correlations *C*_*ij*_ and nonzero pairwise interactions *J*_*ij*_, respectively. It is important to note that, while the *C*_*ij*_ shown in (**b**) take some negative values, the nonzero *J*_*ij*_ shown in (**c**) are all positive. To determine the tipping point of a network using Eq. 1, *J*_*ij*_ > 0 are necessary.

In Fig. 3a, we show the interaction matrix *J*_*ij*_ of the stock network for the 100-day window ending 22 June 1998. Nodes are colour-coded according to the stocks’ various financial sectors. Node size increases with degree, and edge thickness increases with interaction strength. Figure 3b shows the distribution of correlations *P*(*C*_*ij*_), and Fig. 3c shows the distribution of interactions *P*(*J*_*ij*_), for this network. It is important to note that, despite the presence of some negative correlations, the interactions inferred via Graphical Lasso are all positive, allowing us to describe the system with equations of the form of Eq. 1 and find a tipping point which relates to the k-core.

### Mutualism and nestedness

Nestedness quantifies the tendency of the most generalist nodes in a network—those with the most connections— to interact with the most specialist nodes, or those with the fewest connections^2,19–21^. Especially applicable to networks such as ecosystems, this measure posits that the more “nested” a network is (i.e., the more that the generalists interact with the specialists), the more robust it will be.In mutualistic ecosystems, we say that species both providing a benefit to, and reaping some reward from, others in the system are commensalists, while those that only derive a benefit are symbionts. It is shown in^18^ that the commensalists inhabit the maximum k-core of the network and the removal of one, then, has some strong negative impact on an ecosystem. A significant decrease in population of, for example, a pollinator that is a core species could then reverberate throughout a whole ecosystem, as herbivores require enough plant matter as food to maintain their own populations, and these herbivores are then eaten by carnivores.

In the context of real systems, the nestedness of a network has been linked with how mutualistic are the interactions between its members. In Bascompte *et al*. (2003), it is shown that nestedness increases with mutualism in ecological networks, such that highly-mutualistic networks are also highly-nested (as opposed to having a compartmentalised structure)^19^. The system is then organised around a core of interactions, displaying a small number of species interacting with many others and many species which interact only with a few others. Similarly, May *et al*. (2008) noted that, in financial systems, smaller and more specialised banks and businesses tend to have few connexions which are mostly with larger, more "generalist" banks and businesses^36^, thus drawing a comparison between ecology and economics.

**Figure 4:**
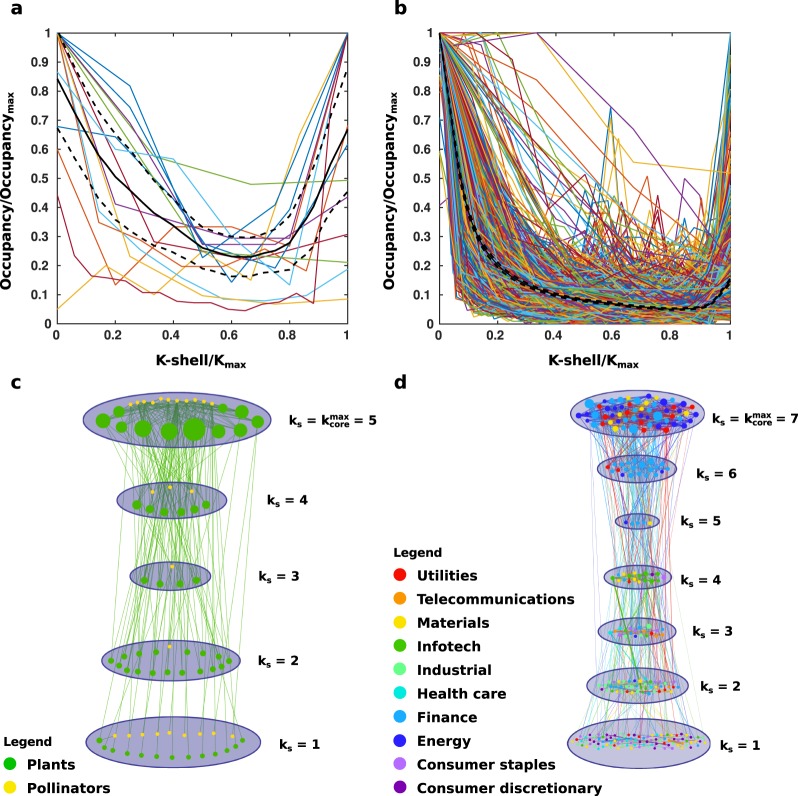
Occupancy of *k*-shells in ecological and financial networks. (**a**) *k*-shell (normalised as $\frac{k-shell}{{k}_{max}}$ versus *k*-shell occupancy (normalised as $\frac{occupanc{y}_{shell}}{occupanc{y}_{max}}$) for the ecological networks; the black solid line gives the average over all ecosystems (N = 15, 95% confidence interval of the average given by the black dashed lines). (**b**) *k*-shell versus occupancy, normalised as in (**a**), for the networks of stocks in the S&P 500 in overlapping 100-day windows ranging between 2 November 2015 and 2 January 1970 (N = 1147; average over all stocks given by the black solid line, 95% confidence interval of the average given by the black dashed lines). (**c**,**d**) show *k*-shell occupancy for a single ecological network^46^ from (**a**) and a single financial network from (**b**) for the 100-day window ending 22 June 1998, respectively. Shell diameter is proportional to occupancy and node size is proportional to degree.

Due to this central core of interactions that is found in mutualistic networks, we hypothesise that the structure and occupancy of the network is related to its robustness and resilience. To study this hypothesis we examine both the ecological and financial networks via k-core decomposition. We probe the structure of the networks using k-shell decomposition and discover an unexpected structure in common among these networks when examining the population of each k-shell. As in Fig. 4, when the occupancy of each shell is plotted for both the ecological and financial networks, we find that the average over all histograms for each set of networks forms a “U” curve rather than the upward-sloping line expected for a random network^18,32,33^. Figure 4a,b show, respectively, normalised histograms of all k-shell occupancies for the 15 ecological and 1147 financial networks as well as the averages over these with a 95% confidence interval. Figure 4c,d show, respectively, examples of k-shell occupancy for the ecological network onstructed using data from Santos *et al*. (2010) and the financial network shown in Fig. 3a. Node size is given by in-degree (Fig. 4c, a directed network) or degree (Fig. 4d, which is undirected). The diameter of the shells is in proportion to the occupancy; it can be seen that the maximum core and outermost shell are the most populated, and some intermediate shell is drastically less populated. This presents as a “U”-shape on the histograms in Fig. 4a,b. By contrast, the ecological networks whose structure do not differ significantly from the random case, do not display a “U”-shape when averaging over histograms of their k-shell occupancies. Similarly, in the case of the financial networks, we find that there are on average more nodes in the lower k-shells than would be expected from the random case, for times both of financial stability and financial crisis.

In random networks, the occupancy of each k-shell increases with *k*, such that the core of the network houses the largest amount of nodes^32,33^. Since the nodes at the core provide structure to the rest of the network, this strengthens the network against external global changes in conditions that lower the interaction strengths of all member nodes^18^—a relevant example in the case of ecological networks would be climate change. In this case, the nodes at the core, which have the most interactions, will persist the longest. Additionally, a larger k-core means there are more highly interconnected nodes providing a “backbone” for those parts of the network less essential to its survival. However, the very same structure also renders the network vulnerable to attacks where a node (or nodes) are removed simply based on some random probabilistic scheme. The bulk of the nodes are located at or near the core of the network, thus going on simple probability alone, it is much more likely that one of these central nodes will be removed by a random attack as compared to one of the nodes at the periphery (of which there are fewer). Such removals will diminish the network’s robustness and eventually cause the network to collapse.

With Eq. 1, we proved that high occupancy of the core provides resilience against global attacks. Moreover, we propose that the “U”-shape structure obtained in the financial and ecological networks, and shown in Fig. 4, contributes to the resiliency of both financial and ecological networks in two ways. First, the increased occupancy of the core (relative to those shells with middling values of *k*) provides structure to the network by the core nodes’ high interaction strengths and thus helps to protect against the effects of external global changes; second, the large amount of nodes in the lowest k-shells provides a measure of protection against random attacks by acting as a buffer. In other words, the more important nodes in the inner shells, whose removal would quickly lead to a collapse of the network, are better-protected because their probability of random deletion decreases when they are surrounded by more nodes whose survival is not essential to that of the network. (For a more in-depth examination of this, please see Supplementary Information, section “Testing the networks”). A larger core provides more stability and thus a more robust network, while a larger amount of nodes in the outermost shells provides greater protection to those essential nodes. In fact,^18^ shows that larger ecosystems with more species in both the core and the periphery are more resilient to attack than ones with fewer species overall. This runs contrary to the diversity-stability paradox proposed by May^3,6,7^, which states that more diverse ecosystems are actually less stable.

As nodes are deleted starting from the outermost shell and approaching the tipping point, the network collapses from the outside in. The *k* = 1 shell will collapse first, with enough nodes removed; the *k* = 2 shell will soon follow if enough of its nodes are removed, and so forth. For ecological networks, the extinction of species in k-shells increasingly close to the tipping point changes the mutualistic structure of the network. As more and more species are removed and the outermost shells collapse, species closer and closer to the core of the network become commensalists (which only receive a benefit from other species) rather than symbionts (which both give and receive a benefit). At some point, the core species are providing too much for the rest of the network and not receiving enough of a benefit in return; as this continues and there cease to be any species providing benefits to the others, the network is rendered unsustainable and totally collapses. Relating to the shell structure of the network, as more and more species are removed, the k-shell structure changes. Eventually, the maximum k-shell (or “core”) *k*_*max*_ takes an integer value less than the tipping point *K*_*γ*_, leading to the collapse of the network.

As *γ* is proportional to the average of interactions in a network^18^, we can estimate *K*_*γ*_ also as $\frac{1}{\gamma }$. We apply such an estimation to the stock market networks. *γ* must take positive values, therefore it is important that the interactions (if not necessarily the correlations) of the stocks in each network take values greater than 0. As with the ecological networks, those nodes in the core and higher-numbered k-shells are integral to the stability of the network, or “keystone” nodes^18,66^. This is analogous to the discovery of "influencers” in social networks, without whom the networks would collapse, using k-shell decomposition^35,40^.

**Figure 5:**
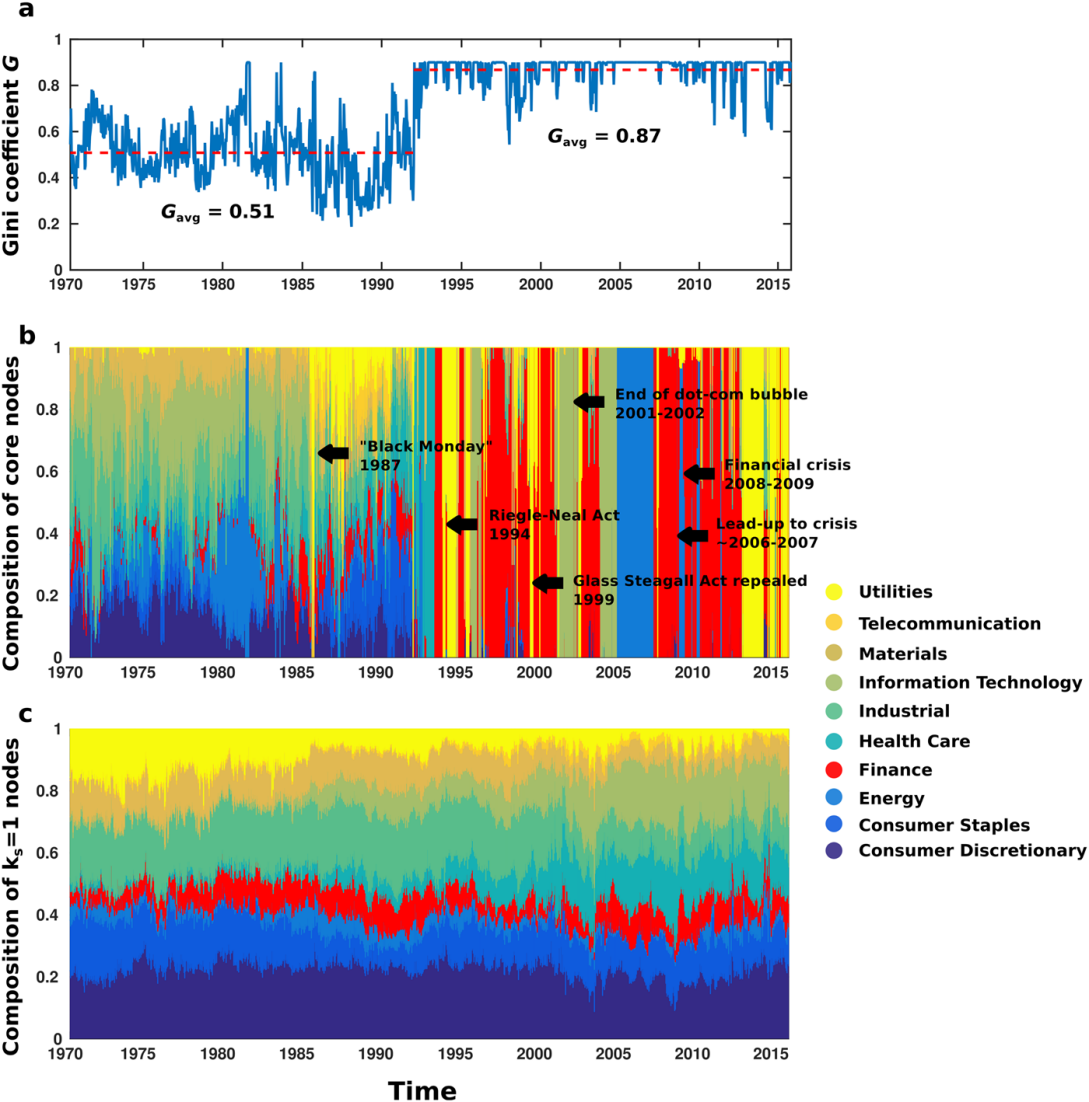
K-shell population of financial networks by economic sector. (**a**) shows the Gini coefficient *G* of each network’s maximum core ${k}_{core}^{max}$. Note the drastic shift from average *G* of 0.51 before 1992 to a much higher average *G* of 0.87 afterwards. This agrees with the shift from a core occupied by a variety of industries to one occupied by only one or two. (**b**) Shows the composition of the maximum core for each network. The sudden transition to domination by one or two sectors could stem from the advent of online trading in the early 1990s allowing for anyone, anywhere to be involved in the stock market, or of financial deregulation in the 1990s. (**c**) shows the composition of the outermost (*k* = 1) shell of the financial networks, which stays more or less constant.

Moreover, examination of real financial data bears out the importance of nodes belonging to the core. Figure 5 shows the Gini coefficient *G* of the core (Fig. 5a), as well as the membership of the ${k}_{core}^{max}$ and the outermost *k* = 1-shell broken down by economic sector (Fig. 5b,c, respectively). The Gini coefficient is a measure of equality in how resources are shared among a population; a higher Gini coefficient implies greater inequality, i.e., a small number of the population controlling the majority of the resources^67^. (Details of the calculation can be found in Supplementary Information). Here, the population is the set of stocks in the S&P 500 while the “resource” is coreness, or membership in the maximum k-core. In 1992, there is a sudden transition from a k-core with more diverse membership among industries (average Gini coefficient *G*_*avg*_ = 0.51 from 1970 to 1992) to a k-core mostly populated by one or two industries (*G*_*avg*_ = 0.87 from 1992 to 2015). Despite this, Fig. 5c shows that the outermost core of the network has remained fairly stable in composition over the past 45 years, with some changes due to, e.g., increasing prevalence of digital devices and the accompanying rise of the information technology sector.

**Figure 6:**
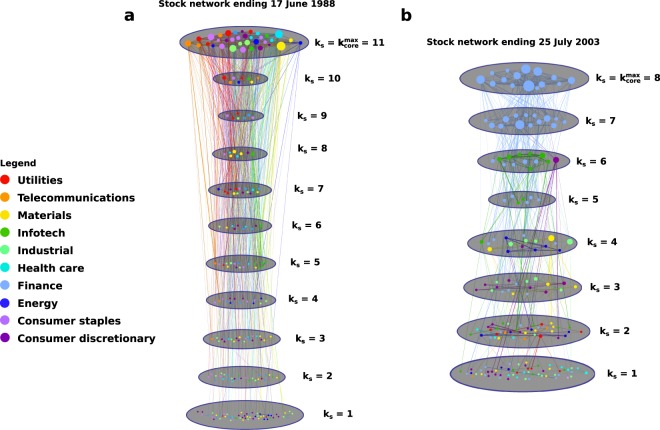
K-shell decomposition of stock market networks before and after 1990s deregulation. (**a**) Pre-deregulation; stock network for 100-day period ending 17 June 1988. The various sectors of the economy are all well-represented in each shell, including the maximum k-core, implying that the economic health of the United States is not dependent upon any single industry. (**b**) Post-deregulation; stock network for 100-day period ending 25 July 2003. The innermost cores of the network are dominated by a single industry (finance), meaning that turmoil in the financial sector would have negative effects on the stock market and the U.S. economy as a whole. Each network is color-coded by economic sector and divided into its constituent shells.

In contrast to this stability is the composition of the ${k}_{core}^{max}$, which seems to be largely dependent upon the market sector(s) moving the United States economy at a given time. We see in Fig. 5b that businesses in the financial sector, coloured red, dominated the ${k}_{core}^{max}$ for nearly two years preceding the 2008–2009 US financial crisis. The demise of these core companies (seen in the lack of any financial-sector stocks in the core during the worst of the crisis) had dire consequences for the US economy. Indeed, a similar sentiment is shown in the idea of businesses being “too big to fail”—there are certain companies whose removal from the economy would cause some catastrophic collapse or near-collapse. The underlying origins of the 2008–2009 US financial crisis can be seen in Fig. 5b, in the sudden shift from a ${k}_{core}^{max}$ which is fairly balanced in composition to one ruled by one or two economic sectors. Figure 6 shows a typical stock network before this transition (Fig. 6a) in contrast with a typical stock network after the transition (Fig. 6b). Note the diversity of industries in the core in Fig. 6a as compared with the dominance of the core by the financial industry in Fig. 6b.

**Figure 7:**
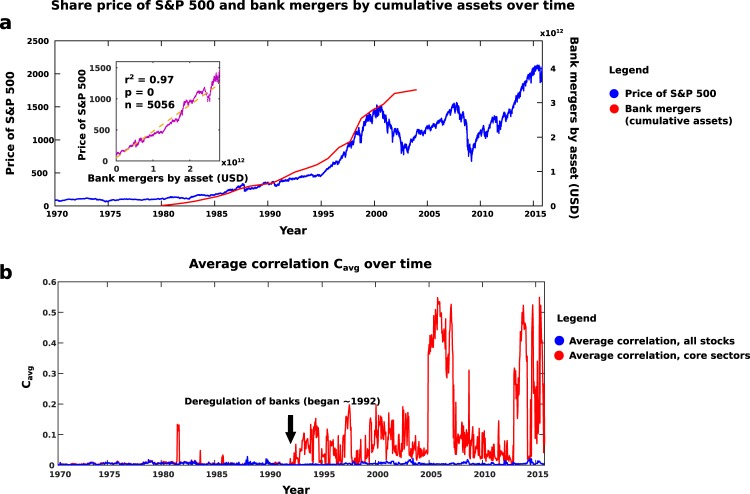
Changes in stock network metrics due to deregulation. (**a**) Average share prices of the S&P 500 index (1970–2015) and cumulative assets in bank mergers (1980–2004), versus time. The deregulation of the mid-1990s allowed banks to acquire one another and merge with impunity, massively increasing the amount of assets involved. Simultaneously, the average share price of the S&P 500 nearly tripled. Once there were no more small banks left to absorb, these mergers and acquisitions dropped off. Inset: increase of assets moved from 1980 to 2000 is strongly correlated with average share price of the S&P 500 (*r*^2^ = 0.97, *p* = 0, *n* = 5056 data points). (**b**) Average correlation between member stocks of the S&P 500 from 1970 to 2015. Following bank deregulation, average correlations of core stocks become much greater than the average over all stocks, most likely due to the makeup of the core becoming drastically more homogeneous once fewer banks had most of the investing power. As in^23^, the correlation between core stocks jumps dramatically higher (i.e., almost lock-step) preceding periods of economic distress such as the Enron scandal 2002–2003 and financial crisis of 2008.

This shift occurred in the early 1990s and was likely the result of a confluence of factors. First, the deregulation of the banks during the administration of President Clinton: the Glass Steagall Act prohibited mergers of investment and lending banks but was toothless by the early to mid-1990s; major banks began to merge as early as 1990, creating institutions which were “too big to fail”^24,25,27,28^. The Riegle-Neal Act of 1994 further loosened the rules by allowing bank acquisitions across state lines^26,28^, greatly increasing the number of banks available to merge or acquire. Although this caused the price of the S&P 500 to nearly triple in value over the next five years as banks merged together at an unprecedented rate (Fig. 7a), it set the stage for an economy whose maximum k-core was dominated, and thus whose health was dictated, by one or two industries (the large blocks of colour seen in Fig. 5b)^24,28^. Only a few banks controlling much of the country’s wealth has led to periods of homogenous k-core. These few banks rush to invest in what industries they deem most promising, but since there are so few banks, there is not a great deal of diversity in the investments and one industry dominates the maximum k-core. Thus, institutions that are “too big to fail” create industries that are “too core to fail”—maximum-core-dominant industries where instability could prove catastrophic for the overall financial health of the country. The financial industry was, for several years, one of these core industries; moreover, the uncontrolled merging of banks had led not only to the loss of diversity in the industries making up the maximum core, but to the reduction of the number of influential companies making up that particular core industry! Sure enough, this confluence of factors meant that the subprime mortgage crisis and subsequent collapse or near-collapse of a few essential banks in 2008 severely hurt the economy of the United States and sent shockwaves through the global economy.

Of note is the fact that, while previous works have experimentally found network structures where there are a large group of peripheral nodes connected to a large group of core nodes, k-shell decomposition offers a network-theoretical method of determining the role of various nodes in the robustness of a network. In this manner, one may, for example, determine the magnitude of harm that a given species’ extinction or even endangerment will do to an ecosystem. Moreover, by dividing the ecosystem into shells, one may determine how dire is the situation of the ecosystem, i.e., if many species in the shell closest to the tipping point are disappearing or in danger of doing so. In the case of the stock market, there are two types of predictors: individual stocks in the core and core-dominant economic sectors (following the deregulation of banks in the early 1990s^24–28^ and the increased prevalence of automated trading systems^29–31^, core nodes tend to belong to only one or two economic sectors out of a possible ten). The individual companies are those that should be monitored to prevent their failure and an ensuing “domino effect” throughout the economy, as happened in the financial crisis of 2008, while the correlation of stocks within the core-dominant sectors should be monitored for signs of the stocks moving in lock-step— a harbinger of market volatility^23^ (Fig. 7b). More generally, one may predict the future health of a network by identifying key nodes, and then act upon those predictions in order to maintain the network’s integrity.

The “U-shape” in the k-shell occupancies is reminiscent of networks with core-periphery structure. These display a highly interconnected subset of nodes at the core and another subset of nodes at the periphery loosely connected to those core nodes^15^. Core-periphery structure has been found in networks ranging from economies^68^ to interactions amongst a troop of monkeys^69^. Such similarities may mean that the U-shape structure seen in the the networks we examined— and its corresponding implications for network robustness— could be more common than previously thought. By k-core theory, the nodes in the outermost shells are loosely connected to the rest of the network (e.g., nodes in the *k* = 1-shell have only one link to the rest of the network following the iterative pruning described earlier, those in the *k* = 2-shell have two, etc.), while those in the maximum k-shell (the core) have the greatest number of links to the rest of the network. A U-shape in the occupancy histogram of k-shells would then mean that the majority of the nodes have either very few links to the rest of the network (those in the outermost shells) or very many links (those in the innermost shells and core)—the type of network defined by core-periphery structure.

### Comparison with diversity-stability paradox of May

The networks examined in the present work which display U-shaped k-shell occupancy histograms not only have higher values of maximum core ${k}_{core}^{max}$, but are also more stable (see the previous section) than random networks of the same number of nodes. For the nonlinear dynamical systems described by Equations 1, the stability of the fixed-point solution given by Eq. 2 is controlled by a Jacobian matrix ${M}_{ij}({\overrightarrow{x}}^{\ast })$^18^:

5 $${M}_{ij}({\overrightarrow{x}}^{\ast })=\frac{\delta {\dot{x}}_{i}}{\delta {x}_{j}}{| }_{\overrightarrow{x}={\overrightarrow{x}}^{\ast }}$$

Here, species *i* and *j* have densities *x* and fixed-point solutions x^*^. It is found that the nontrivial fixed-point solution is stable if the eigenvalues of Eq. 5 have a negative real part and that the solution becomes unstable when the system collapses (that is, the threshold on mutualistic benefit ${K}_{\gamma }={k}_{core}^{max}$, where ${k}_{core}^{max}$ is the maximum k-shell, or “core” of the network found following a k-shell decomposition); the details of the calculation are in^18^. As stated in the section “Network Dynamics”, analytical solution of the dynamical equations of a mutualistic system finds that more diverse systems, i.e., those with more species at the maximum core of the network under a k-shell decomposition, are more resilient to global and random local attacks. The finding that a larger maximum k-core number ${k}_{core}^{max}$ makes the system more stable is one of the key points of Morone, Del Ferraro, and Makse (2019)^18^. A higher value of ${k}_{core}^{max}$ implies a greater number of species present in the system, as ${k}_{core}^{max}$ can take a value of, at maximum, one less than the number of species found to be at the *k*_*max*_-core of the network (corresponding to each of such species having an interaction with all other members of the core). Ecosystems with fewer species are thus bound by a lower maximum possible value of ${k}_{core}^{max}$ and are less stable.

This runs opposite to the conclusion of Sir Robert May, who proposed that *increasing* the diversity of ecosystems, counterintuitively, decreases their stability (the “diversity-stability paradox”)^3^. Here, the difference lies in the choice of stability matrix; the one used by May does not depend upon the fixed-point solution^3,18^:

6 $$M{\prime} =-{\delta }_{ij}+\frac{{A}_{ij}}{{K}_{\gamma }}$$

Again, *i* and *j* refer to species, but the adjacency matrix *A*_*ij*_ is a random model and thus $M{\prime}$ is random as well^3^. The stability condition here also requires that the eigenvalues of this matrix have negative real parts. The maximum eigenvalue of the stability matrix in^18^ is upper-bounded by ${\lambda }_{max}^{M}=-\,\frac{\gamma }{{K}_{\gamma }+1}$, guaranteeing that ${\lambda }_{max}^{M} < 0$ and the nontrivial fixed point is stable. However, the stability condition in^3^ requires that the largest eigenvalue of the adjacency matrix *A*_*ij*_ is upper-bounded as ${\lambda }_{max}^{A} < {K}_{\gamma }$. In Equation 6, ${\lambda }_{max}^{A}$ is a nondecreasing function of the number of species^3,18^, meaning that the more species there are in an ecosystem, the more difficult it is to satisfy this stability condition. The conclusion of Morone, Del Ferraro, and Makse (2019), in contrast, depends upon the fixed-point solution and finds that for increasing values of species in the maximum core (and thus increasing values of ${k}_{core}^{max}$), the stability condition is actually easier to satisfy^18^. Hence, the diversity-stability paradox is done away with.

## Discussion

Network robustness is an issue relevant to many systems the world over, from finance to the Internet to ecosystems; the well-being of economies, countries, and even the earth depend upon the survival of these networks so it is important to quantify their resilience. A number of methods have thus been proposed previously to calculate robustness. Similarly important is the point at which enough nodes have been removed to cause network collapse. If the tipping point of collapse of an ecosystem (for example) is known, prevention of that collapse will be more possible because we will see warning signs in the degrading structure of the network. Here, we have proposed a new k-core-based robustness that is based in theory rather than individual examinations of discrete networks, and a method of determining a network’s tipping point of collapse which is also based in a k-shell decomposition of a network. Since the maximum k-core is a topological invariant (i.e., structurally based and not dependent upon, for example, how the nodes are labelled)^18^, this method can be generalised across networks so long as they have all positive interactions.

Following k-shell decompositions of both ecological and financial networks, we find that histograms of the occupancy of each k-shell form a “U-shape.” Thus, the outermost shells— the periphery— and the innermost shells— the core— are the most highly-populated, while the intermediate shells are very sparsely populated. This is, in fact, similar to core-periphery structure, wherein there exists a subset of nodes (the “core”) connected strongly not only to each other, but to most of the other nodes in the network, and a subset of nodes (the “periphery”) connected more loosely to a few of the nodes in the core^70^. Core-periphery structure has been found in many types of networks, from world systems, to economics, to social networks among primates^15,71,72^. Our structure differs in that there are more than two subsets, or shells, but the underlying idea remains: there is a central subset of nodes which are integral for the maintenance of network structure, while the most peripheral nodes are less important for the network’s survival and may cease to provide a mutualistic interaction without causing a total collapse of the network.

It is known that the degree of a node in a random network is highly correlated with the k-shell to which the node belongs, as noted in previous work by Kitsak *et al*.^35^ and found in many other studies in the literature. Crucially, however, we observe that the U-shape cannot be found in a random network and thus cannot be directly related to the degree distribution. There may be some higher-order degree correlations which determine the non-random U-shape, and although that is beyond the scope of this paper, it would be an interesting problem to directly relate the k-core structure to these higher-order correlations. We note that the k-core is composed of nodes of degree at least *k* that are also surrounded by other nodes with degree at least *k*, clearly indicating both strong degree-degree correlations and that these correlations could be responsible also for the U-shaped distribution.

The persistence of the core and inner shells, essential for network structure, even in the face of average interactions being weakened across the whole network shows the importance of the “U”-shaped k-shell structure to those networks’ survival, and to determining and predicting networks’ health. An abundance of species in the outer shells protects those species in the maximum core and inner shells when the ecosystem comes under attack, though when the maximum-core species cease to provide any mutualistic benefit to the other species in the network, the ecosystem cannot sustain itself and collapses fully. It can be useful to monitor the populations and interactions of species in a given ecosystem through the lens of such a k-core decomposition: those in the outer shells, often commensalists who derive more benefits than they provide, tend to disappear or cease to provide mutualistic benefits first with less of a negative impact; the network collapses when symbionts, who have interactions with many other species both in the core and at the periphery, disappear or cannot provide enough of a mutualistic benefit for the system to be sustainable. Thus, certain species may be treated as indicators of the health of a given ecosystem in that a population decline could be an early warning of a collapse if corrective action is not taken.

Similarly, the businesses and industries whose stocks make up the maximum core of a financial network could be viewed as indicators of economic health. That is, if certain core businesses begin to show signs of distress via their stock activity, they could be more closely regulated in order to prevent (or mitigate the effects of) a future recession or even depression. Since the bank deregulation of the 1990s, the banks, emboldened by deregulation, merged repeatedly until there were only a few major banks remaining; this was the advent of banks that were “too big to fail,” as they were responisble for the money of much of the United States. Much of the investing power was now in the hands of a few banks, leading to less diversity in where the money was going (as shown by the sudden change during the 1990s to a core dominated by only one or two industries) and thus tying the health of the economy to the fortunes of companies in, e.g., finance or information technology. Furthermore, the adoption of automated online trading systems has led to further homogenisation of investing strategies, where trades are made using computerised analysis of past data, leading to similar behaviour among everyone from investment firms and banks to individuals^29–31^. This similar behaviour then becomes the past data that is analysed and used for decision-making, creating a cycle of increasing dependence upon a few companies in one or two industries (as seen in Fig. 5b) that can lead to economic turmoil if several of these companies go under. It has been shown^23^ that stocks move in lock-step (high correlation between returns) prior to drastic swings in the market; we find that this lock-step behaviour is shown even more dramatically within the core-dominant economic sectors. Beyond single core companies, entire core sectors may be monitored for signs of abruptly and abnormally high correlation, and corrective or preventative measures may then be taken.

The idea of monitoring a core subset of vital nodes in order to preserve the structure of a network is also generalisable beyond the applications explored in this paper. Due to the breadth of networks which display a core-periphery structure, one may use the k-core structure of a social network to more effectively inoculate populations against disease, or take advantage of the structure of Internet networks to better guard digital infrastructure against viral attacks. In sum, since core-periphery and k-shell structure are ubiquitous in real-world networks, a better understanding of what makes these networks robust— and how to determine whether or not that robustness is being endangered— can be utilised to protect against, or at least soften the effects of, a wide range of variously catastrophic events.

## Methods

### Data acquisition

Ecological data was downloaded from the Interaction Web DataBase^45^, for anemone-fish; plant-pollinator; plant-ant; and plant-disperser interactions. These data were converted to a standard format of a binary directed adjacency matrix where a value of "1” indicates a link (or interaction) and a value of 0 denotes no interaction. We selected 49 plant-pollinator networks (further details in Supplementary Information).

The economic data was daily open and close prices for stocks belonging to the S&P 500 index over a period spanning from 2 January 1970 to 5 November 2015. Data of each individual stock were only used for the time during which the stock belongs to the index. A formal list of changes to the composition of the S&P 500 was consulted^63^ to ensure that the networks include the correct stocks. Data for stocks that were still extant at the time of data collection (5 November 2015) were collected from the Yahoo! Finance page^64^ and data for stocks that were no longer being traded were purchased in order to avoid survivorship bias^65,73^.

We then calculated the daily log-returns *r*_*i*_(*t*) of a given stock *i* for the period *t* during which it belongs to the index as follows^74^:

7 $${r}_{i}(t)={\rm{\log }}\,(\frac{Clos{e}_{i}(t)}{Ope{n}_{i}(t)})$$

Here, the variables *Close*_*i*_(*t*) and *Open*_*i*_(*t*) represent the opening and closing prices of the stocks on the same day. Open-to-close return data maintains synchronicity in the trading hours of all the stocks^5,75^. Some stocks, for example, might be traded online during hours when the New York Stock Exchange is closed, whereas others might not be (and would not have been, prior to the advent of online trading); this could potentially lead to a situation in which one stock belonging to the S&P 500 for some duration, is traded more than another stock belonging to the index for the same amount of time.

## Supplementary information

Supplementary Information.

## Data Availability

The datasets produced in this study will be made available upon reasonable request. Requests should be sent to the corresponding author.

## References

[1] Domínguez-García, V. & Muñoz, M.A. Ranking species in mutualistic networks. *Sci. Rep*., **5** (2015). 10.1038/srep08182 PMC4313099 25640575

[2] Bascompte J, Jordano P (2007). Plant-Animal Mutualistic Networks: The Architecture of Biodiversity. Annu. Rev. Ecol. Evol. Syst..

[3] May RM (1972). Will a Large Complex System be Stable?. Nature.

[4] Mantegna RN (1999). Hierarchical structure in financial markets. Eur. Phys. J. B.

[5] Bury, T. Collective behaviours in the stock market – a maximum entropy approach. *arXiv Ph.D Thesis* arXiv:1403.5179v2 (2014).

[6] Haldane AG, May RM (2011). Systemic risk in banking ecosystems. Nature.

[7] May RM (1982). Mutualistic interactions among species. Nature.

[8] Caldarelli, G. & Vespignani, A. Large Scale Structure and Dynamics of Complex Networks: From Information Technology to Finance and Natural Science (World Scientific, Singapore) (2007).

[9] Morone, F. & Makse, H. A. Influence maximization in complex networks through optimal percolation. *Nature* **524**, 65–68 (2015). 10.1038/nature14604 26131931

[10] Morone F, Roth K, Min B, Stanley HE, Makse HA (2017). A model of brain activation predicts the neural collective influence map of the human brain. Proc. Natl. Acad. Sci.

[11] Arese, F., Del Ferraro, G., Sigman, M. & Makse, H. A. How the brain transitions from conscious to subliminal perception. *Neuroscience* **411**, 279 (2019). 10.1016/j.neuroscience.2019.03.047 PMC6612454 31051216

[12] Kim Y, Chen YS, Linderman K (2015). Supply network disruption and resilience: A network structural perspective. J. Oper. Manag..

[13] Cohen R, Erez K, ben-Avraham D, Havlin S (2000). Resilience of the Internet to Random Breakdowns. Phys. Rev. Lett..

[14] Zhang X, Martin T, Newman MEJ (2015). Identification of core-periphery structure in networks. Phys. Rev. E.

[15] Borgatti SP, Everett MG (2000). Models of core/periphery structures. Soc. Net.

[16] Verma T, Russmann F, Araújo NAM, Nagler J, Herrmann HJ (2016). Emergence of core-peripheries in networks. Nature Comm.

[17] Bollobás, B. & Riordan, O. Percolation. (Cambridge University Press, Cambridge, 2006).

[18] Morone, F., Del Ferraro, G., & Makse, H.A. The k-core as a predictor of structural collapse in mutualistic ecosystems. *Nature Phys.* **15**, 95–102 (2019). 10.1038/s41567-018-0304-8 PMC12674604 41346342

[19] Bascompte J, Jordano P, Melián CJ, Olesen JM (2003). The nested assembly of plant-animal mutualistic networks. Proc. Natl. Acad. Sci..

[20] Beckett, S. J., Boulton, C. A. & Williams, H. T. P. FALCON: a software package for analysis of nestedness in bipartite networks *F1000Res*. **3** (2014). 10.12688/f1000research.4831.1 PMC4244763 25485095

[21] Johnson S, Domínguez-García V, Muñoz MA (2013). Factors Determining Nestedness in Complex Networks. PLOS One.

[22] Atmar, W. & Patterson, B. D. The measure of order and disorder in the distribution of species in fragmented habitat. *Oecologia* 96, 373–382 (1993). 10.1007/BF00317508 28313653

[23] Ramkumar, A. Warning Sign: Markets Moving in Lockstep. *The Wall Street Journal*, https://www.wsj.com/articles/markets-moving-in-lockstep-are-latest-warning-sign-1540382401 (2018). Accessed 24 October 2018.

[24] Carroll, L. Glass-Steagall repeal had nothing to do with financial crisis, http://www.th-o-meter/statements/2015/aug/19/bill-clinton/bill-clinton-glass-steagall-had-nothing-do-financi/ (2015). Accessed 25 August 2018.

[25] Rhoades, S. A.* Bank Mergers and Industrywide Structure, 1980–94*, Staff Study 179 (Board of Governors of the Federal Reserve System, Washington, D.C.) (1996).

[26] Pilloff, S. J.* Bank Merger Activity in the United States, 1994–2003*, Staff Study 176 (Board of Governors of the Federal Reserve System, Washington, D.C.) (2004).

[27] Carpenter, D. H. & Murphy, M. M.* Permissible Securities Activities of Commercial Banks Under the Glass-Steagall Act (GSA) and the Gramm-Leach-Bliley Act (GLBA)*, CRS Report for Congress (Congressional Research Service, Washington, D.C.) (2010).

[28] Sherman, M.* A Short History of Financial Deregulation in the United States* (Center for Economic and Policy Research, Washington, D.C.) (2009).

[29] Automated Trading. History of Trading Systems, http://www.automatedtrading.com/2014/01/13/history-trading-systems/ (2014). Accessed 17 December 2018.

[30] Automated Trading. What is a Trading System, http://www.automatedtrading.com/2013/12/19/trading-system/ (2013). Accessed 17 December 2018.

[31] Zuckerman, G., Levy, R., Timiraos, N. & Banerji, G. Behind the Market Swoon: The Herdlike Behavior of Computerized Trading. *The Wall Street Journal*, https://www.wsj.com/articles/behind-the-market-swoon-the-herdlike-behavior-of-computerized-trading-11545785641 (2018). Accessed 26 December 2018.

[32] Dorogovtsev SN, Goltsev AV, Mendes JFF (2006). k-Core Organization of Complex Networks. Phys. Rev. Lett..

[33] Dorogovtsev SN, Goltsev AV (2008). Critical phenomena in complex networks. Rev. Mod. Phys..

[34] Min, B., Morone, F. & Makse, H. A. Searching for influencers in big-data complex networks (*Diffusive Spreading in Nature, Technology, and Society*, Bunde, Caro, Karger, & Vogl, eds.). (Springer Verlag, 2016).

[35] Kitsak M (2010). Identification of influential spreaders in complex networks. Nature Phys.

[36] May RM, Levin SA, Sugihara G (2008). Ecology for bankers. Nature.

[37] Holland JN, DeAngelis DL, Bronstein JL (2002). Population dynamics and mutualism: functional responses of benefits and costs. Am. Nat..

[38] Bastolla U (2009). The architecture of mutualistic networks minimizes competition and increases biodiversity. Nature.

[39] Thébault E, Fontaine C (2010). Stability of Ecological Communities and the Architecture of Mutualistic and Trophic Networks. Science.

[40] Pei, S., Morone, F. & Makse, H. A. Theories for influencer identification in complex networks. In Spreading Dynamics in Social Systems (Lehmann & Ahn, eds.) (Springer Nature, 2007).

[41] Alon, U. An Introduction to Systems Biology: Design Principles of Biological Circuits (CRC Press, 2006).

[42] Gao J, Barzel B, Barabási A-L (2016). Universal resilience patterns in complex networks. Nature.

[43] Amit, D. J. Modeling Brain Function: The World of Attractor Neural Networks (Cambridge University Press, 1989).

[44] Glass L, Kauffman SA (1973). The logical analysis of continuous, non-linear biochemical control networks. J. Theor. Biol..

[45] Interaction Web DataBase. Resources, https://www.nceas.ucsb.edu/interactionweb/resources.html Accessed 21 May 2017.

[46] Santos GMM, Aguiar CML, Mello MAR (2010). Flower-visiting guild associated with the Caatinga flora: trophic interaction networks formed by social bees and social wasps with plants. Apidologie.

[47] Blúthgen N, Stork NE, Fiedler K (2004). Bottom-up control and co-occurrence in complex communities: honeydew and nectar determine a rainforest ant mosaic. Oikos.

[48] Beehler B (1983). Frugivory and polygamy in birds of paradise. The Auk.

[49] Snow, B. K. & Snow, D. W.* Birds and Berries* (Poyser Monographs, 1988).

[50] Bartomeus I, Vilá M, Santamaria L (2008). Contrasting effects of invasive plants in plant-pollinator networks. Oecologia.

[51] Arroyo MTK, Primack RB, Armesto JJ (1982). Community studies in pollination ecology in the high temperate Andes of Central Chile. I. Pollination mechanisms and altitudinal variation. Amer. J. Bot..

[52] Clements, R. E. & Long, F. L. Experimental pollination. An outline of the ecology of flowers and insects (Carnegie Institute of Washington, 1923).

[53] Elberling H, Olesen JM (1999). The structure of a high latitude plant-flower visitor system: the dominance of flies. Ecography.

[54] Inouye DW, Pyke GH (1988). Pollination biology in the Snowy Mountains of Australia: comparisons with montane Colorado, USA. Aust. J. Ecol..

[55] Kaiser-Bunbury CN, Memmott J, Múller CB (2009). Community structure of pollination webs of Mauritian heathland habitats. Perspectives in Plant Ecology, Evolution and Systematics.

[56] Kato M, Makutani T, Inoue T, Itino T (1990). Insect-flower relationship in the primary beech forest of Ashu. Kyoto: an overview of the flowering phenology and seasonal pattern of insect visits. Contr. Biol. Lab. Kyoto Univ.

[57] Kevan, P. G. High arctic insect-flower visitor relations: the inter-relationships of arthropods and flowers at Lake Hazen, Ellesmere Island, Northwest Territories, Canada. Ph.D. thesis, University of Alberta (1970).

[58] McCullen CK (1993). Flower-visiting insects of the Galapagos Islands. Pan-Pac. Entomol..

[59] Memmott J (1999). The structure of a plant-pollinator food web. Ecol. Lett..

[60] Friedman J, Hastie T, Tibshirani R (2008). Sparse inverse covariance estimation with the graphical lasso. Biostat.

[61] Mazumder R, Hastie T (2012). Exact Covariance Thresholding into Connected Components for Large-Scale Graphical Lasso. J. Mach. Learn. Res..

[62] S&P Dow Jones Indices, http://us.spindices.com/indices/equity/sp-500 Accessed 5 November 2015.

[63] Siblis Research. Article Archive, http://siblisresearch.com/data/ Accessed 11 May 2017.

[64] Yahoo Finance. Business Finance, Stock Market, Quotes, News, https://finance.yahoo.com Accessed 5 November 2015.

[65] Norgate Data. Premium Data: Historical Data, https://www.premiumdata.net/products/premiumdata/ushistorical.php Accessed 11 May 2017.

[66] Berry D, Widder S (2014). Deciphering microbial interactions and detecting keystone species with co-occurrence networks. Front. Microbiol.

[67] Gini, C.* Variability and Mutability* (C. Cuppini, Bologna, 1912).

[68] Krugman, P.* The Self Organizing Economy* (Wiley-Blackwell, 1996).

[69] Corradino C (1990). Proximity structure in a captive colony of Japanese monkeys (Macaca fuscata fuscata): An application of multidimensional scaling. Primates.

[70] Csermely P, London A, Wu LY, Uzzi B (2013). Structure and dynamics of core/periphery networks.

[71] Orsini C, Gregori E, Lenzini L, Krioukov D (2014). Evolution of the Internet k-Dense Structure. IEEE/ACM Transactions on Networking.

[72] Saavedra S, Reed-Tsochas F, Uzzi B (2008). Asymmetric disassembly and robustness in declining networks. Proc. Natl. Acad. Sci..

[73] Investopedia. Survivorship Bias, http://www.investopedia.com/terms/s/survivorshipbias.asp Accessed 17 April 2017.

[74] Bonanno G, Lillo F, Mantegna RN (2000). High-frequency Cross-correlation in a Set of Stocks. Qual. Fin..

