# Supplementary material for: K-core robustness in ecological and financial networks
Source: Sci Rep. 2020 Feb 25;10:3357. doi: 10.1038/s41598-020-59959-4 (PMC7042264; doi:10.1038/s41598-020-59959-4)
Supplement: Supplementary file 1 — Supplementary Information. [file 41598_2020_59959_MOESM1_ESM.pdf]

K-core robustness in ecological and financial networks

Kate Burleson-Lesser[a,b], **Flaviano Morone**[a], **Maria S. Tomassone**[c], **and Hernán A. Makse**[a,1]

[a]City College of New York, Levich Institute
[b]The Graduate Center at the City University of New York
[c]Rutgers University, Department of Chemical and Biochemical Engineering
[1]To whom correspondence should be addressed. E-mail: hmakse@ccny.cuny.edu

ABSTRACT

In many real-world networks, the ability to withstand targeted or global attacks; extinctions; or shocks is vital to the survival of the network itself, and of dependent structures such as economies (for financial networks) or even the planet (for ecosystems). Previous attempts to characterize robustness include nestedness of mutualistic networks or exploration of degree distribution. In this work we present a new approach for characterizing the stability and robustness of an ecosystem (plant-pollinator interactions) by studying the distribution of the k-shell of the underlying network. We find that high occupancy of nodes in the inner and outer k-shells and low occupancy in the middle shells of financial and ecological network (yielding a "U-shape" in a histogram of k-shell occupancy) provide resilience against both local targeted and global attacks. Investigation of this highly-populated core gives insights into the nature of a network (such as sharp transitions in the core composition of the stock market from a mix of industries to domination by one or two in the mid-1990s) and allow predictions of future network stability, e.g., by monitoring populations of "core" species in an ecosystem or noting when stocks in the core-dominant sector begin to move in lock-step, presaging a dramatic move in the market. Moreover, this "U-shape" recalls core-periphery structure, seen in a wide range of networks including opinion and internet networks, suggesting that the "U-shaped" occupancy histogram and its implications for network health may indeed be universal.

Supplementary Information

Deviation of ecological networks from random case

To make sure that the ecological networks from the Interaction Web DataBase[1] would yield significant results, we generate 10000 random networks of the same size and degree distribution as each real network, and perform a k-shell decomposition on each. We then calculate the p-value as the number of times out of 10000 that k_{max}^{rand} (the maximum k-shell of each generated network) is greater than or equal to k_{max}^{real} (the maximum k-shell of the real network):

$$p = \frac{\# k_{max}^{rand} \geq k_{max}^{real}}{10000} \tag{1}$$

We consider those networks with a p-value less than 0.01 to be significantly different from the random case. This method also culls the networks that are too small to yield very significant results for our purposes (i.e., 10 or 15 nodes). Furthermore, the networks with too high a p-value do not display the "U"-shaped curve when histograms are plotted of their k-shell occupancy, confirming that the significant networks differ from what might be expected in a random case.

A second test that we perform is a series of random attacks on each network, starting with the original adjacency matrix each time and removing successively larger amounts of nodes. The networks previously designated "significant" not only decay more slowly in comparison to their corresponding Erdos-Renyi graphs (generated by randomly reshuffling the original networks while retaining a bipartite structure and the original degree distribution), but specifically the core and those k-shells closest to it are more resilient, persisting even when half the nodes have been attacked and removed. For the corresponding Erdos-Renyi graphs obtained via reshuffling, the latter trend is not as strongly displayed.

Correlation matrices

To construct the correlation matrices of the stocks, we choose windows of 100 days, with a 10-day shift. After investigating multiple windows lengths from 10 days to 500 days, we plotted metrics of the resulting networks over all 45 years, such as the average correlation C_{avg} or the mean log-returns in each time window. We judged that the 100-day windows did not

over-smooth the time series (thus cutting out potentially valuable information) but also did not preserve an excessive amount of noise, as happened in the smaller time windows with smaller shifts. To build the networks in each window, we first subtract the mean of each stock's log-returns in that window:

$$R_i(t) = r_i(t) - \frac{\sum_t^T r_i(t)}{T} \tag{2}$$

where T is the window length, and then compute the Pearson correlations C_{ij} between these new log-returns R of stocks i and j as follows:

$$C_{ij} = \frac{\sum_t R_i(t) R_j(t)}{\sqrt{\sum_t R_i(t)^2 \sum_t R_j(t)^2}} \tag{3}$$

We get 1147 such networks. Stocks which do not belong to the S&P 500 for the entirety of a particular time window are not included in the network for that time window. Because the S&P 500 index always has roughly 500 members[2], the network sizes in each time window generally fluctuate only slightly depending upon how many stocks entered or left the index during that period, ranging from 469 to 498 with an average size of 487. Out of all the networks, there are 10 which have an abnormally low size (around 450 nodes), which can be attributed to a high turnover of stocks in the index during that time (stocks generally enter or leave in the middle of a time window, rendering them ineligible for consideration in that window's network).

Some useful metrics for each network, several of which were mentioned earlier, are the network size N; average correlation $C_{avg}(t)$:

$$C_{avg}(t) = \frac{\sum_i^N \sum_{j \neq i}^N C_{ij}}{N(N-1)} \tag{4}$$

and the average log-returns in that time window $R_{avg}(t)$:

$$R_{avg}(t) = \frac{\sum_i^N \sum_t^T R_i(t)}{TN} \tag{5}$$

Interaction matrices

For the financial data, the matrices C_{ij} can potentially show spurious correlations which arise not from similarities between the behaviours of two stocks, but from the similarity of each to a third stock. As an example, let us imagine three stocks A, B, and D. A and B do not behave in a similar fashion to each other, but they behave similarly (in two different ways) to D. Despite this, A and B would have a high value of correlation $C_{A,B}$, thus implying that they behave alike when they, in fact, do not[3].

Figures 1a and 1b show the correlation and interaction matrices, respectively, for one selected network from the stock data. The stocks have been organized by the economic sectors to which they belong (as indicated by the white boxes); we also verify that the clusters found via a standard clustering algorithm[4] match well with these sectors.

To find the true pairwise interactions J_{ij}, we turn to the Graphical Lasso ("Glasso") algorithm of Friedman, Hastie, and Tibshirani[5,6]. Glasso works by maximizing the penalized log-likelihood over inverse covariance matrices Θ (our "J_{ij}") which are definite and nonnegative:

$$\log\det\Theta - tr(S\Theta) - \lambda\|\Theta\|_1 \tag{6}$$

where tr is the trace; S is the empirical covariance matrix; λ is the penalty; and $\|\Theta\|_1$ is the sum of absolute values of the elements of Θ, or the L_1 norm. The interaction matrices returned by this method are sparse, and values between -10^{-4} and 10^{-4} are set to zero. In Figure 1, for example, we see that many of the interactions are equal to zero though the interactions were nonzero in Fig. 1. The pairwise "correlations" in these cases had arisen from the fact that two stocks' behaviours may each have had similarities to different aspects of the behaviour of a third stock but not to each other[3].

There has been some discussion over the best way to choose a penalty λ, which can present a difficulty to those wanting to use the Graphical Lasso. We follow the method of Mazumder and Hastie[6], which entails finding the threshold at which a giant connected component emerges in the correlation matrix C_{ij} and setting the penalty equal to this value. To find the threshold at which the giant connected component emerges, we use a standard method for percolation of the giant component[7,8]. Testing

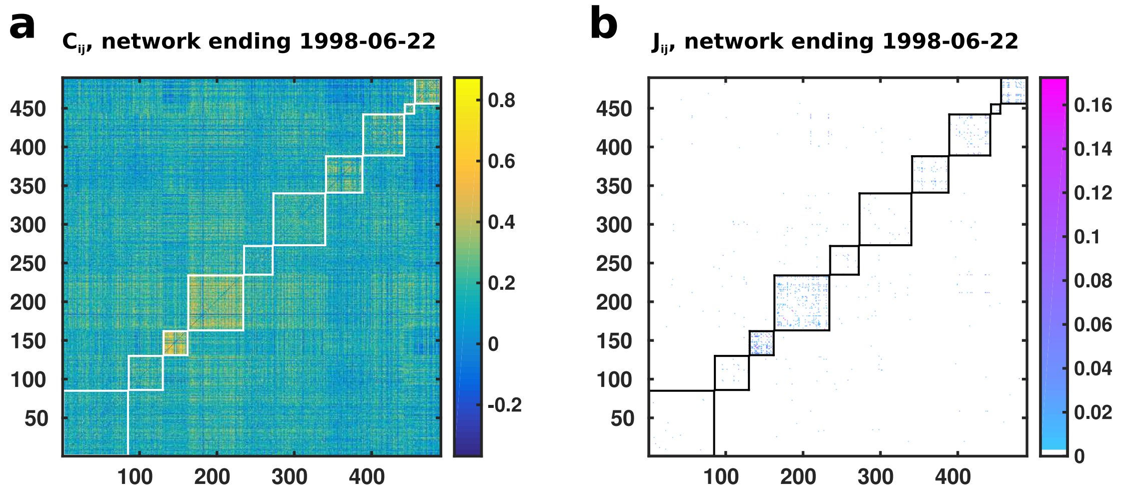

Figure 1. Example network of the S&P 500. (a) The pairwise correlations C_{ij} of a 489-member example stock network over a 100-day window ending 22 June 1998 and **(b)** the corresponding network of interactions J_{ij} for the same network. Note the relative sparseness of the matrix in **(b)** as compared to that in **(a)**. Also of note is that, while the pairwise correlations in **(a)** range approximately from -0.4 to 1.0, the nonzero interactions in **(b)** are all positive, with a maximum at 0.16. The stocks are clustered, as indicated by the boxes, according to the economic sectors to which they belong. Clusters found via a standard clustering algorithm[4] match closely with these sectors.

this for one-third of our networks against a second method of penalty optimisation which involves percolation of the giant component in the interaction matrix J_{ij} over different values of λ—the optimal penalty here is the value at which the giant component emerges—we find that the two methods yield the same, or very similar, results, with an average error of 0.82%. Moreover, we find that the structures of C_{ij} at the percolation threshold and J_{ij} at the optimal lambda are almost exactly the same, with an average structural difference over all 1147 networks of only 0.29% of the nodes.

Despite many of the correlation matrices C_{ij} containing a small amount of negative values, the nonzero entries in all of our interaction matrices J_{ij} are positive. This is important to the structure of the network, as explained in the main text: mutualistic networks and the calculation of K_γ require all-positive interactions[9].

K-shell calculation

To learn about the natures of both the ecological and stock networks, we investigate their structures using the k-shell algorithm[10–12]. To discover which nodes are in which k-shell, we use an iterative search with the following steps:

1. Find all nodes with degree $k = 1$.
2. Remove these nodes and their links from the network.
3. Since there may now be new nodes that have degree $k = 1$, repeat steps **1** and **2** until there are no more nodes with degree $k = 1$ in the network; these nodes belong to the $k = 1$ shell, or $k_s = 1$.
4. Repeat the previous three steps for increasing values of k until all nodes in the network have been categorised as belonging to a k-shell.
5. Those nodes belonging to the innermost k-shell—k_{max}—are considered as belonging to the “k-core” of the network.

As discussed in the Results section, when examining the occupancy of the k-shells for both the ecological and stock networks, rather than shell occupancy which increases with increasing values of k as we would expect from a random network, we find high occupancy in both the outermost (with lower values of k) shells and the innermost (with higher values of k) shells. Minimum occupancy is reached in one of the middle shells.

In the case of the ecological networks, those nodes in the k_{max}-core and other high-numbered k-shells contain the "symbionts," or species that provide some benefit at the same time as they benefit from the actions of other species, and those shells in the outermost shells ($k = 1$ and other low-numbered k-shells) contain the "commensalists," or species which only gain some benefit from the other species but do not provide anything in return. In more general terms, the nodes representing the symbionts are those which give structure to the network, while the nodes representing the commensalists are those which protect the network from destruction due to random attack.

The structure of a random network would render it more susceptible to destruction via random attack, due to a higher probability of attacking nodes in the core which are vital to the structure of the network. For the ecological and stock networks showcased here, these nodes in the core are protected by an increased number of nodes in the outer shells. In these cases, a random attack is less probable to remove a node in the core and more probable to remove one of the nodes in the outer shells.

Nestedness

One way in which we can describe the structure of interactions in a network is the nestedness. In ecological networks, this is the propensity of more specialized species, or those which interact with very few others, to interact with the more generalist species, or those species which have many interactions in the network[13–15]. The more a network displays this property, the more nested it is said to be; in adjacency graph representations of such networks, the graph appears to fill the upper or lower triangle[13, 14, 16–18]. There are several methods of quantifying nestedness; one of the best-known is the "temperature" defined by Atmar and Patterson and implemented in their "Nestedness Calculator" software[18], which draws an analogy between the ecological network and a physical system by describing the "disorder" of the network. Since greater nestedness implies greater order in the interactions between species in the network, more nested systems have lower temperatures. Those systems that are maximally-nested (i.e., temperature $T = 0$) will tolerate no reordering of interactions, whereas less-nested systems can maintain the same degree of system order even if there is some shuffling of interactions[14, 16].

The software implementation works by first reorganizing an $m \times n$ matrix such that the species are arranged from most generalist in their interactions, to most specialist, thus (ideally) filling the upper triangle. An isocline of "perfect nestedness" is then determined for the matrix. A quantity "unexpectedness" or U is then calculated, with the rule that the lack of an interaction before the isocline, or the presence of an interaction beyond the isocline, is "unexpected"[18]. The higher is the value of U for the interaction matrix, the higher is the disorder (and thus the temperature T); ultimately, the temperature is scaled between 0 and 100. It is important to note that Bascompte et al. normalised the temperature as $T' = \frac{100-T}{100}$, thereby scaling the nestedness between 0 (minimal nestedness) and 1 (maximal nestedness)[14]. Figure 2 shows a nested system (Fig. 2a)[19] with such an isocline vs. a nonnested system (Fig. 2b)[20]; indeed, the system in Fig. 2a displays the U-shaped k-shell occupancy histogram and is therefore more robust than the system in Fig. 2b, which does not.

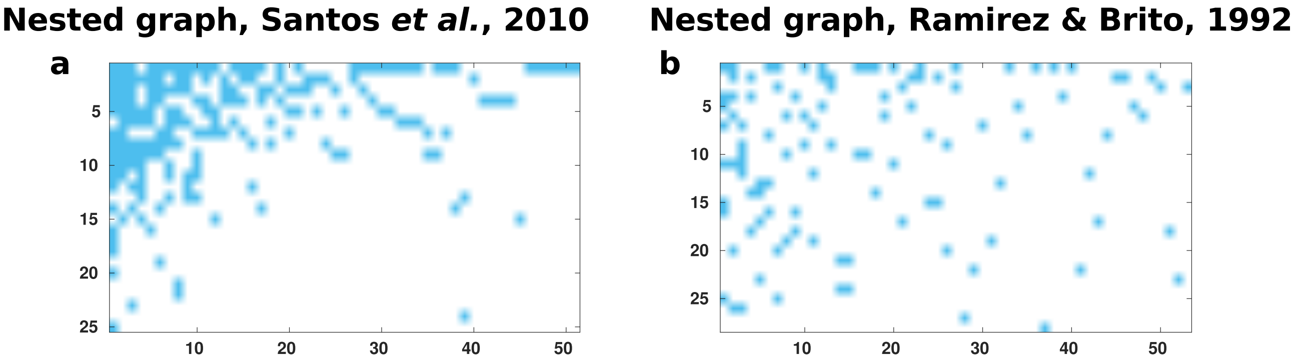

Figure 2. Nestedness in systems with and without a U-shaped k-shell occupancy histogram. (a) A nested system with a well-defined upper-triangular structure. This system (the one in[19]) also has a U-shaped k-shell occupancy histogram and is thus more resilient to local and global attack than the system in **(b)** (from[20]), which has neither a U-shaped k-shell occupancy histogram nor an isocline of nestedness.

A second method of calculating nestedness, which does not employ an isocline and thus does not demand that the matrix structure be rearranged to be as close as possible to upper-triangular, is that of Johnson, Domínguez-García, and Muñoz[15].

Here, the expected nestedness η of a bipartite matrix is calculated as:

$$\eta = \frac{n_1 \langle k^2 \rangle_2 + n_2 \langle k^2 \rangle_1}{\langle k \rangle_1 \langle k \rangle_2 (n_1 + n_2)^2} \quad (7)$$

where n_1 and n_2 are the numbers of nodes in each set of the bipartite matrix (for the case of the ecological networks, these sets would be "plants" and "animals"); k is the number of edges; and $\langle \cdot \rangle_i$ is an average over set i. η takes values which increase with increasing order (nestedness), with the minimum value of η being 1 for a totally random network.

Testing the networks

We test each ecological network using a series of random attacks, to determine how the k-shell structure of each network responds to the successive removal of nodes. To randomly attack the network, we remove from the original network increasing fractions of nodes, such that most of the network remains after the initial attacks and very little of the original network remains after the later attacks. We then observe the size of, for example, the giant connected component or each k-shell following each random attack.

We then compare these results with the effects of random attacks on Erdos-Renyi networks obtained by randomly reshuffling the original ecological networks while retaining their bipartite structures and degree distributions as well as the network sizes. The real ecological networks which display the U-shape in histograms of k-shell occupancy last much longer than their Erdos-Renyi (ER) counterparts. This could imply that networks which have the majority of their nodes in both the outermost and innermost k-shells are more robust as compared to networks with few nodes in the outermost k-shells and the majority in the core, as in random ER networks. The results of random attacks on an example ecological network[19] as opposed to randomised ER networks of the same size, and with edges drawn according to the same degree distribution, as the real network are shown in Figure 3. Fig. 3a shows the persistence of the giant connected component ($G(q)$) for both the real and randomised networks under random removal of a fraction of nodes q. The giant connected component survives considerably longer for the real network (until approximately 80% of the nodes are removed) than for the ER case (until approximately 60% of the nodes are removed). Figs. 3b and 3c show how the k-cores are populated as increasing fractions of nodes are removed and serve to highlight the importance of the core nodes—both networks collapse much more quickly when the nodes being removed are chosen from the maximum or innermost cores. Note that here, we show the populations of each k-core, i.e., a given k-shell and all of the shells contained within it. Again, the real network (Fig. 3b) lasts in general longer than its ER counterpart (Fig. 3c).

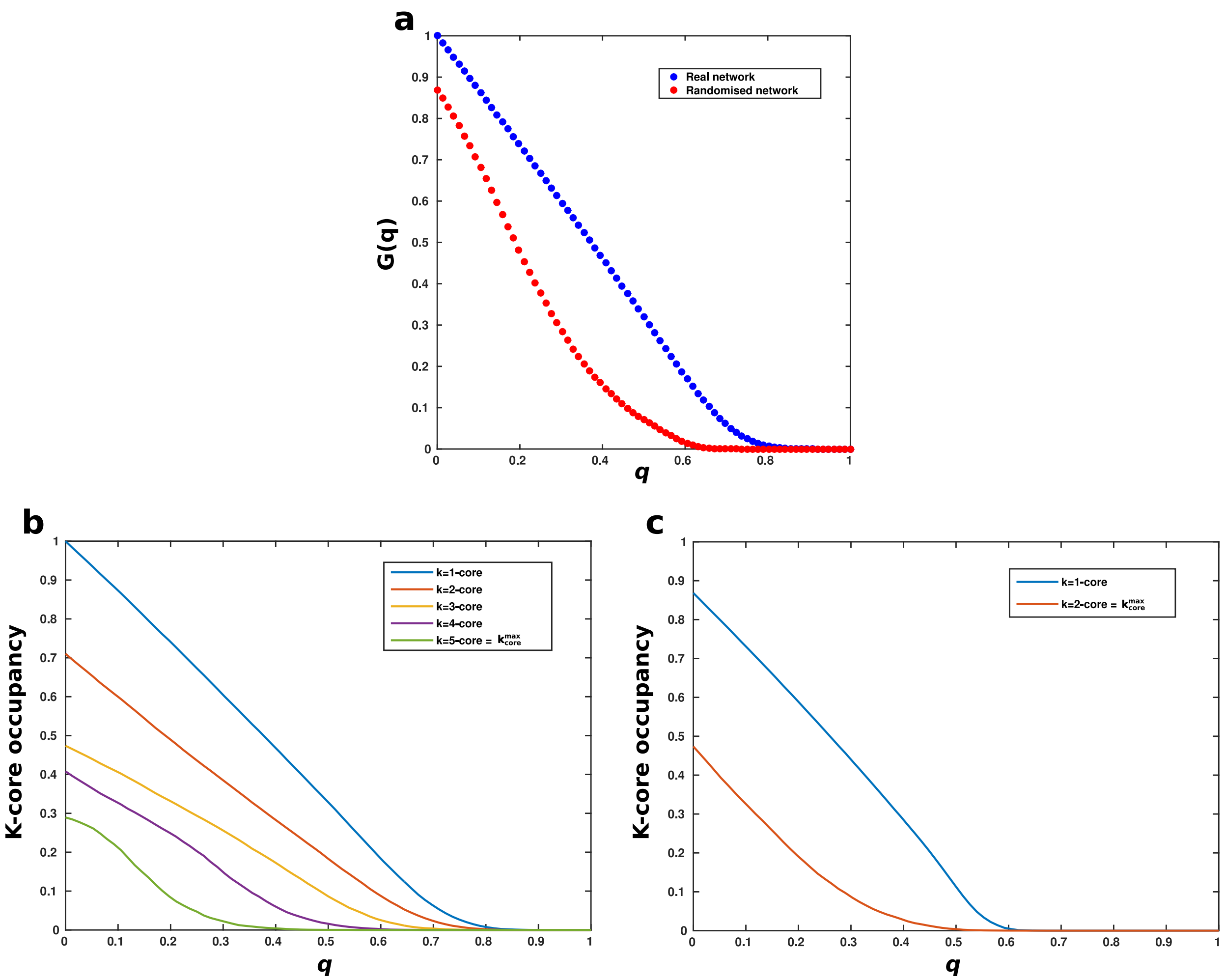

Figure 3. Deviation of behaviour of real ecological network from random ER networks of the same size and degree distribution under random attack. (a) The giant component of the real ecological network (blue dots) persists for longer than the random case (red dots) under random removal of nodes. $G(q)$ denotes the percentage of nodes in the network belonging to the giant component following random removal of a fraction q of nodes. For the random case, the giant component has collapsed at $q \approx 0.62$, while for the real network, the giant component collapses around $q \approx 0.83$. **(b)** and **(c)** show, respectively, the species remaining in each k-core after a removal of some fraction q of nodes from a real example network (Santos *et al.*, 2010) and a random Erdos-Renyi network of the same dimensions and average degree. The real network, shown in **(b)**, survives much longer (to $q \approx 0.8$) under random deletion of nodes than does the random network shown in **(c)** (to $q \approx 0.6$).

Interestingly, in the case of the financial networks, we note that networks constructed during times of relative financial stability (Fig. 4a, 95% confidence intervals) have on average more nodes in the outer and medial shells k-shells than do those constructed during times of financial crisis or recession[21] (Fig. 4b, 95% confidence intervals). This agrees with the notion that the nodes in the outer and medial shells die off first to protect the nodes in and near the core, which are integral to the structure and stability of the network.

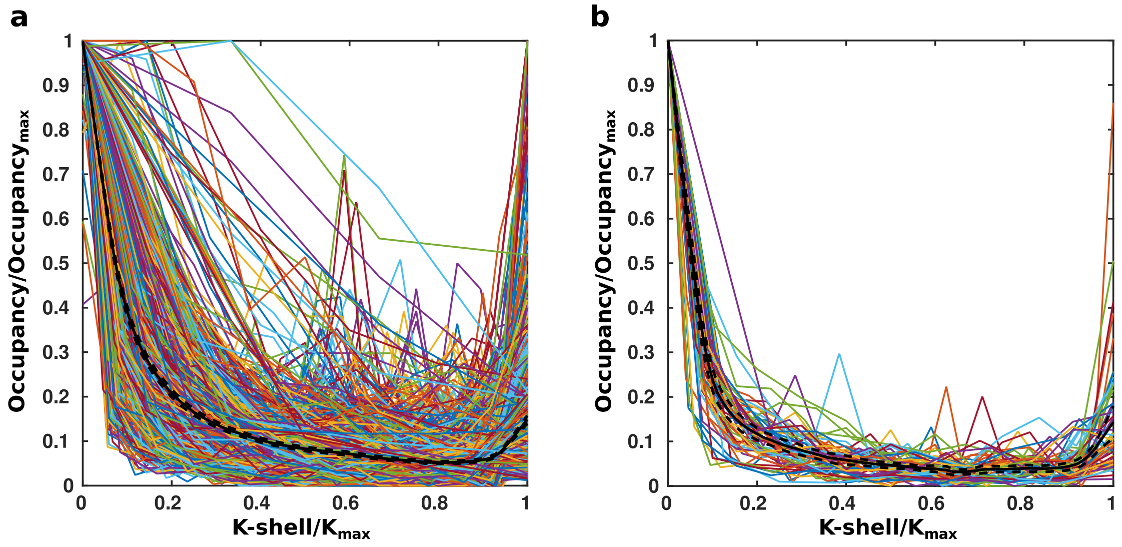

Figure 4. Comparison of k-shell population during stable and unstable periods in finance. (a) Histogram of stock market k-shell occupancy during relatively stable periods, i.e., not periods of notable financial turmoil (N = 1086, average given by black solid line, 95% confidence intervals given by dashed lines, normalised as in Fig. 4a and 4b of the main text). There are more nodes on average in the lower and middle k-shells, aside from the outermost shell, as compared to **(b)** the networks during recession or crash periods (N = 61, average given by black solid line, 95% confidence intervals given by dashed lines, normalised as in Fig. Fig. 4a and 4b of the main text). The networks in **(b)** may be viewed as systems "on the brink," not actually collapsed but dangerously close. The nodes in the lower shells disappeared first, protecting the core nodes important for the stability of the network, while the average occupancy of the core remains basically unchanged.

Gini coefficient

The Gini coefficient G is a measure of statistical disperion originally developed by Corrado Gini to measure income inequality[22]; it may, however, be used more generally to quantify the level of inequality within a population for a given "resource." It is calculated as:

$$G = \frac{\sum_{i=1}^{n} \sum_{j=1}^{n} |x_i - x_j|}{2n \sum_{i=1}^{n} x_i} \tag{8}$$

for a population of size n where member i has some quantity of resources x_i. Higher levels of inequality result in higher Gini coefficients and for sufficiently large n, a totally unequal population may acheive a Gini coefficient of $G = 1$[22].

For the purposes of this paper, we implement the Gini coefficient to measure the inquality of "coreness" among the various financial sectors making up the stock market. In practice, this means that the domination of the core by only one or two sectors would yield a much higher Gini coefficient than a core made up of all or most of the various sectors. Indeed, we see a sharp transition from a lower average Gini coefficient ($G = 0.51$ from 1970 to 1992) to a much higher average value ($G = 0.87$ from 1992 to 2015) that coincides with the change from a k-core occupied by a range of stocks from different economic sectors, to a k-core dominated by one or two sectors.

References

1. Interaction Web DataBase. Resources. URL https://www.nceas.ucsb.edu/interactionweb/resources.html. Accessed 21 May 2017.
2. S&P 500. S&P Dow Jones Indices. URL http://us.spindices.com/indices/equity/sp-500. Accessed 5 November 2015.
3. Bury, T. Collective behaviours in the stock market – a maximum entropy approach. *arXiv Ph.D Thesis* arXiv:1403.5179v2 (2014).

4. Le Martelot, E. & Hankin, C. Fast multi-scale detection of relevant communities in large-scale networks. *Comput. J.* **56** (2013).

5. Friedman, J., Hastie, T., & Tibshirani, R. Sparse inverse covariance estimation with the graphical lasso. *Biostat.* **9**, 432–441 (2008).

6. Mazumder, R. & Hastie, T. Exact Covariance Thresholding into Connected Components for Large-Scale Graphical Lasso. *J. Mach. Learn. Res.* **13**, 781–794 (2012).

7. Gallos, L., Sigman, M., & Makse, H.A. The conundrum of functional brain networks: small-world efficiency or fractal modularity. *Front. Physiol.* **3** (2012).

8. Hu, Y., Havlin, S., & Makse, H.A. Conditions for viral influence spreading through multiplex correlated social networks. *Phys. Rev. X* **4**, 021031 (2014).

9. Morone, F., Del Ferraro, G., & Makse, H.A. The k-core as a predictor of structural collapse in mutualistic ecosystems. *Nature Phys.* URL https://doi.org/10.1038/s41567-018-0304-8 (2018).

10. Dorogovtsev, S.N., Goltsev, A.V., & Mendes, J.F.F. *k*-Core Organization of Complex Networks. *Phys. Rev. Lett.* **96**, 040601 (2006).

11. Dorogovtsev, S.N. & Goltsev, A.V. Critical phenomena in complex networks. *Rev. Mod. Phys.* **80**, 1275–1335 (2008).

12. Min, B., Morone, F., & Makse, H.A. Searching for influencers in big-data complex networks (*Diffusive Spreading in Nature, Technology, and Society*, Bunde, Caro, Karger, & Vogl, eds.). (Springer Verlag, 2016).

13. Bascompte, J. & Jordano, P. Plant-Animal Mutualistic Networks: The Architecture of Biodiversity. *Annu. Rev. Ecol. Evol. Syst.* **38**, 567–593 (2007).

14. Bascompte, J., Jordano, P., Melián, C.J., & Olesen, J.M. The nested assembly of plant-animal mutualistic networks. *Proc. Natl. Acad. Sci.* **100**, 9383–9387 (2003).

15. Johnson, S., Domínguez-García, V., & Muñoz, M.A. Factors Determining Nestedness in Complex Networks. *PLOS One* **8**, 1-7 (2013).

16. Domínguez-García, V. & Muñoz, M.A. Ranking species in mutualistic networks. *Sci. Rep.* **5** (2015).

17. Beckett, S.J., Boulton, C.A., & Williams, H.T.P. FALCON: a software package for analysis of nestedness in bipartite networks *F1000Res.* **3** (2014).

18. Atmar, W. & Patterson, B.D. The measure of order and disorder in the distribution of species in fragmented habitat. *Oecologia* **96**, 373–382 (1993).

19. Santos, G.M.M., Aguiar, C.M.L., & Mello, M.A.R. Flower-visiting guild associated with the Caatinga flora: trophic interaction networks formed by social bees and social wasps with plants. *Apidologie* **41**, 466–475 (2010).

20. Ramirez, N. & Brito, Y. Pollination biology in a palm swamp community in the Venezuelan Central Plains. *Bot. J. Linn. Soc.* **110**, 277–302 (1992).

21. Wikipedia. List of stock market crashes and bear markets. URL https://en.wikipedia.org/wiki/List_of_stock_market_crashes_and_bear_markets. Accessed 18 April 2017.

22. Gini, C. *V*ariability and Mutability (C. Cuppini, Bologna, 1912).
